# A novel miR-200b-3p/p38IP pair regulates monocyte/macrophage differentiation

**DOI:** 10.1038/celldisc.2015.43

**Published:** 2016-01-26

**Authors:** Xiao Yu, Qi-Long Wang, Yue-Fang Li, Xu-Dong Wang, Anlong Xu, Yingqiu Li

**Affiliations:** 1 Key Laboratory of Gene Engineering of the Ministry of Education, State Key Laboratory of Biocontrol, School of Life Sciences, Sun Yat-Sen University, Guangzhou, China

**Keywords:** miR-200b-3p, monocyte/macrophage differentiation, p38IP

## Abstract

Monocyte/macrophage differentiation represents a major branch of hematopoiesis and is a central event in the immune response, but the molecular mechanisms underlying are not fully delineated. Here we show that p38 mitogen-activated protein kinase (MAPK) interacting protein (p38IP) is downregulated during monocyte/macrophage differentiation *in vitro*. Overexpression of p38IP halted monocyte/macrophage differentiation, whereas forward knockdown of p38IP by RNA interference induced G1/S arrest and promoted monocyte differentiation into macrophages and the maturation of macrophages as well. Moreover, we found that miR-200b-3p was upregulated during monocyte/macrophage differentiation and mediated the downregulation of p38IP by binding to the 3′ untranslated terminal region of p38IP mRNA. Overexpression of a miR-200b-3p mimic resembled the effect of p38IP knockdown, whereas a miR-200b-3p inhibitor blocked monocyte/macrophage differentiation by enhancing p38IP expression. Further western blotting analysis revealed that p38IP downregulation enhanced the activity of p38 MAPK and the subsequent accumulation of cyclin-dependent kinase inhibitor p21, thus promoting G1/S arrest and monocyte/macrophage differentiation. Moreover, the decline of GCN5 acetyltransferase caused by p38IP downregulation was required but was not sufficient for monocyte/macrophage differentiation. This study demonstrated a new role for p38IP and a novel miR-200b-3p/p38IP pair in the regulation of monocyte/macrophage differentiation.

## Introduction

The human p38 mitogen-activated protein kinase interacting protein (p38IP) was first identified in a yeast two-hybrid screening as the binding partner of p38 mitogen-activated protein kinase (NCBI Accession #AF093250); it contains 733 amino-acid residues with a calculated molecular mass of 80 kD. In the hematopoietic system, p38IP was predicted to be a novel transcriptional regulator in human CD34 antigen-positive hematopoietic cells [1]. The expression of p38IP was downregulated in prostate carcinoma [2]. During mouse gastrulation, p38IP was found to be critical for the downregulation of E-cadherin, which is required for the completion of the epithelial–mesenchymal transition (EMT), by binding and activating p38 [3]. By using mass spectrometry, p38IP was found to be a mammalian protein homologous to yeast Spt20, which is a specific component of the Spt-Ada-Gcn5 acetyltransferase (SAGA) coactivator complex and was demonstrated to be required for the assembly and integrity of the SAGA complex [4, 5]. Additionally, p38IP participated in the regulation of endoplasmic reticulum stress by binding to the promoter of endoplasmic reticulum stress-induced genes [6], and it was shown to mediate starvation-induced autophagy through the regulation of mammalian Atg9 trafficking [7]. Furthermore, we demonstrated that p38IP regulates G2/M progression, spindle formation and hypoacetylation of α-tubulin by suppressing ubiquitination-induced degradation of GCN5 [8]. However, it is not known whether and how p38IP functions in hematopoiesis.

Macrophages, which were first discovered by Élie Metchnikoff for their ability to phagocytose invading pathogens, are well known for their pivotal roles both in innate and adaptive immunity and have critical roles in tissue development, homeostasis and repair [9, 10]. The majority of macrophages are end-stage mature cells that are developed from hematopoietic stem cells through bone marrow myeloid progenitor cells and then monocytes. Multiple stage-specific transcription factors, cell cycle factors and signaling regulatory proteins are involved in monocyte/macrophage (MO/MΦ) differentiation [9]. Dysregulation of MO/MΦ differentiation can lead to various diseases, such as inflammation, autoimmune disease and cancer.

In recent years, microRNAs (miRNAs) have emerged as key players in cell development, including hematopoietic cell development [11, 12]. The rapid increase in knowledge of miRNAs has also provided new opportunities for clinical diagnosis and therapy [13, 14]. miRNAs are a class of regulatory, noncoding and single-stranded RNAs with lengths of approximately 22 nucleotides. miRNAs can have vital roles in a wide variety of biological processes by repressing protein expression at the posttranscriptional level, primarily through base-pairing with the 3′ untranslated terminal region (UTR) of the target genes, which in turn results in cleavage or the repression of translation [15]. In addition to providing a global transcriptional control mechanism, miRNAs can fine-tune gene expression by effecting more subtle and rapid changes, which is thought to occur during hematopoietic cell development [16–18]. Some miRNAs are limited to certain stages in development or certain tissues and cell types and thus having important roles in cell-specific development [19, 20]. During the stage of MO/MΦ differentiation, several miRNAs have been reported to be important regulators, such as miR-155, miR-424, miR-142-3p and miR-223 [21–24]. Further identification and characterization of the miRNAs that are involved in hematopoietic cell differentiation would advance our understanding of this area.

In the present study, we found that miR-200b-3p was upregulated during MO/MΦ differentiation and targeted the p38IP 3′UTR for posttranscriptional regulation. This effect led to the downregulation of p38IP mRNA and thus the p38IP protein. Downregulation of p38IP during MO/MΦ differentiation induced G1/S arrest and then promoted MO/MΦ differentiation in a p38/p21-dependent pathway. During the differentiation, p38IP downregulation induced a decline of GCN5 expression, which contributed to MO/MΦ differentiation but was not sufficient for this process. Collectively, our findings revealed that p38IP and miR-200b-3p are novel and crucial players in the regulation of MO/MΦ differentiation.

## Results

### p38IP downregulation during MO/MΦ differentiation

**Figure 1:**
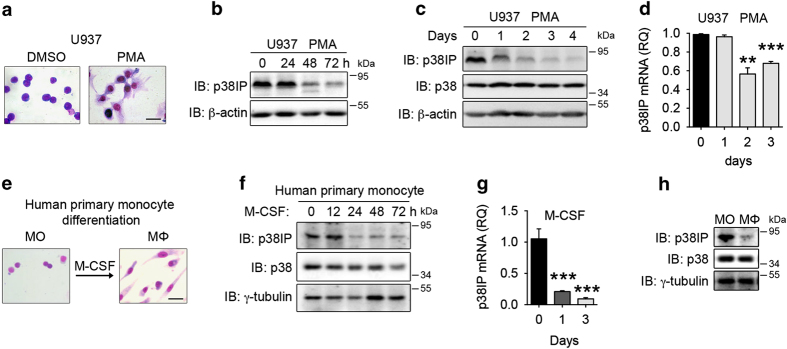
Downregulation of p38IP during monocyte differentiation. (**a**–**d**) Time-dependent downregulation of p38IP in U937 cells with phorbol myristate acetate (PMA) stimulation. U937 cells were treated with PMA for the indicated times to induce differentiation. The cells were stained by Wright–Giemsa staining, and the morphology was analyzed by microscopy at ×100 magnification; a representative field is shown (**a**). The scale bar represents 20 μm. The cells were collected for western blotting analysis of p38IP protein (~83 kDa) expression (**b**, **c**) or quantitative PCR (qPCR) analysis of p38IP mRNA expression (**d**). Human primary monocytes were differentiated into macrophages by culturing with recombinant human macrophages colony-stimulating factor (M-CSF) for 7 days. The cells were then stained by Wright–Giemsa staining, and the morphology was analyzed by microscopy at ×100 magnification; a representative field is shown (**e**). The scale bar represents 20 μm. In addition, the cell lysates were used for western blotting analysis of p38IP protein expression (**h**). Human peripheral blood monocytes were cultured with M-CSF to induce differentiation for the indicated times, and the cells were harvested for western blotting analysis of p38IP protein expression (**f**) or qPCR analysis of p38IP mRNA expression (**g**). The scale bars represent the means±s.e.m. (*n*=3). **P<0.01, ***P<0.001, versus controls. The data are representative of at least three independent experiments with similar results.

The human monoblastic leukemia cell lines U937 and THP-1 have been used as models for *in vitro* MO/MΦ differentiation studies [25, 26]. When U937 cells were treated with phorbol myristate acetate (PMA) to induce MO/MΦ differentiation (Figure 1a), we observed that the levels of p38IP protein declined in a time-dependent manner (Figures 1b and c). Quantitative real-time PCR confirmed a twofold decrease in p38IP mRNA levels (Figure 1d). These results indicated that p38IP was downregulated during U937 differentiation. Similar results were observed in THP-1 cells (Supplementary Figures S1a and b). Furthermore, we treated HL-60 cells with VD_3_ and U937 cells with ATRA and found that the p38IP mRNA and protein levels decreased during these two treatments (Supplementary Figures S1c–f), which indicated that the downregulation of p38IP is a general phenomenon in the process of differentiation.

To determine whether such downregulations occur in primary human cells, we purified human peripheral blood CD14^+^ monocytes and differentiated them into macrophages with human macrophage colony-stimulating factor (M-CSF) *in vitro*. Successful differentiation was verified morphologically with Wright–Giemsa staining (Figure 1e) and by immune surface marker CD14 detection by fluorescence-activated cell sorter (Supplementary Figure S1g). As expected, the levels of p38IP protein and mRNA continued to decrease with differentiation (Figures 1f and g) and maintained at an extremely low expression level in macrophages (Figure 1h). Similar results were obtained by using M-CSF to differentiate mouse primary monocytes into macrophages (Supplementary Figure S1h). Taken together, these observations suggest that monocyte differentiation into macrophages is associated with a decrease in p38IP expression.

### p38IP blocks monocyte differentiation into macrophages

**Figure 2:**
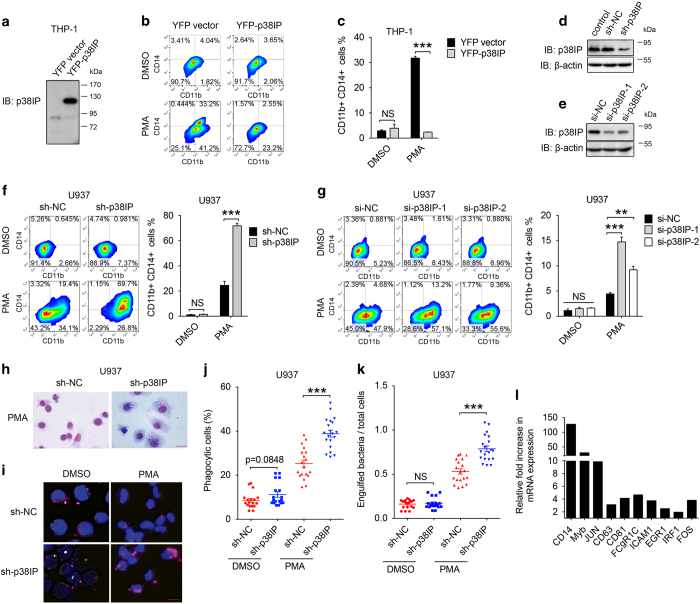
p38IP negatively regulates monocyte differentiation and function. (**a**–**c**) THP-1 cells were transfected with yellow fluorescent protein (YFP)-tagged p38IP or empty vector, followed by phorbol myristate acetate (PMA) stimulation. The overexpression of p38IP was verified by western blotting (**a**), and CD11b/CD14-positive cells were detected by flow cytometry. Representative fluorescence-activated cell sorting (FACS) analysis and percent values of CD11b/CD14-positive cells are shown in (**b**, **c**). (**d**–**g**) p38IP was knocked down by short hairpin RNAs (shRNAs) or small interference RNAs (siRNAs) in U937 cells for 48 h, and the cells were then stimulated with PMA (dimethyl sulfoxide (DMSO) treatment serves as a negative control) for 48 h. The knockdown efficiency was determined by western blotting (**d**, **e**), and CD11b/CD14-positive cells were detected by flow cytometry. Representative FACS analysis and percent values of CD11b/CD14-positive cells are shown (**f**, **g**). (**h**) Morphological analysis of U937 sh-NC and sh-p38IP cells. The cells were exposed to PMA for 48 h and then stained by Wright–Giemsa staining. A ×630 magnification of a representative field is shown. The scale bar represents 20 μm. (**i**–**k**) U937 cells (sh-NC, sh-p38IP) were exposed to 10 nm PMA for 48 h (DMSO treatment serves as a negative control) and incubated with labeled *E. coli* bacteria for 1 h. A representative field of phagocytic activity is shown in (**i**). The scale bar represents 20 μm. Twenty fields of view were selected randomly, and both phagocytic and total cells were counted. The ratio was measured and is shown in (**j**). Engulfed bacteria and total cells were counted, and the ratio is shown in (**k**). (**l**) Relative fold increase in mRNA expression levels of differentiation-related genes in p38IP knockdown cells. Control (sh-NC cells)=1. The scale bars represent the means±s.e.m. (*n*=3). **P<0.01, ***P<0.001 compared with control groups. All data are representative of at least three independent experiments with similar results. NS, not significant.

Because p38IP was downregulated during MO/MΦ differentiation, we hypothesized that p38IP may act as a negative regulator of MO/MΦ differentiation. To confirm this hypothesis, we overexpressed p38IP in THP-1 cells (Figure 2a) and then induced differentiation with PMA. We then analyzed the percentage of cells that were positive for CD11b and CD14, which are markers of MO/MΦ differentiation. Consistent with our hypothesis, the percentage of CD11b/CD14-positive cells dramatically decreased following the overexpression of p38IP (Figures 2b and c). Similar results were obtained in U937 cells (Supplementary Figures S2a and b). Moreover, when we silenced p38IP expression in U937 cells by using p38IP-mRNA-specific short hairpin RNAs (shRNAs) (sh-p38IP) or small interference RNAs (siRNAs) (si-p38IP) or using scrambled shRNA (sh-NC) or siRNA (si-NC) sequences as negative controls (Figures 2d and e), an increase in CD11b/CD14-positive cells was observed in the p38IP knockdown cells after differentiation (Figures 2f and g). To determine whether the downregulation of p38IP positively regulated the maturation and function of monocyte-derived macrophages, we compared the morphology and phagocytic function of differentiated U937 sh-NC and sh-p38IP cells. As expected, sh-p38IP-transfected U937 cells exhibited a more mature differentiation phenotype, including a larger size, increased chromatin condensation, decreased nuclear/cytoplasmic ratio and more vacuoles (Figure 2h). In addition, after incubation with mcherry-conjugated *Escherichia coli*, there was an increase of approximately 20% in the number of mature macrophage cells that engulfed bacteria (Figures 2i and j). Moreover, the number of engulfed bacteria per cell also increased in sh-p38IP-transfected U937 cells (Figure 2k), thus indicating the maturation of these macrophage. Similar results were obtained in THP-1 cells (Supplementary Figures S2c–h). We further sequenced and compared the transcription levels of genes in both differentiated control cells and p38IP-knockdown cells. The results indicated that, in addition to CD14, many MO/MΦ differentiation-related genes exhibited higher mRNA expression levels after knockdown of p38IP (Figure 2l). These data demonstrated that p38IP blocks the differentiation of monocytes undergoing differentiation into macrophages.

### Targeting the p38IP 3′UTR by miRNA-200b-3p

We next considered the mechanism that contributed to the downregulation of p38IP during differentiation. Given that (i) microRNAs have been reported to participate in MO/MΦ differentiation, (ii) miRNAs can both inhibit mRNA translation and target mRNA for degradation [27], and (iii) in our experiments, both the p38IP mRNA level and protein levels were downregulated, we sought to determine whether miRNAs may be involved in the downregulation of p38IP.

First, we used two bioinformatic algorithms to predict the miRNAs that may target p38IP. By using the TargetScanHuman and StarBase databases, we searched for the miRNAs with a putative target sequence in the 3′UTR of the gene encoding p38IP (FAM48A) and found at least 15 miRNAs that may interact with the p38IP 3′UTR (data not shown).

**Figure 3:**
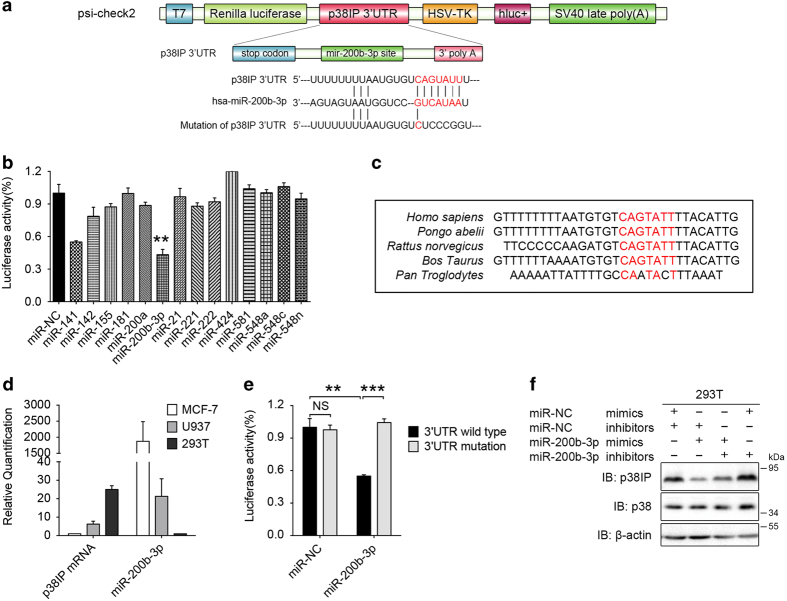
miR-200b-3p directly binds with the p38IP 3′ untranslated terminal region (UTR). (**a**) Schematic representation of the reporter constructs. The Renilla luciferase-coding region was transcribed under the control of the T7 promoter and the luc of the HSV-TK promoter. The sequences shown below indicate the putative miR-200b-3p target site on the 3′UTR wild-type allele, the mutated derivative (mutation) and the pairing regions of miR-200b-3p. (**b**) Luciferase reporter assays of 293T cells. The cells were co-transfected with the reporter plasmid carrying the p38IP 3′UTR (construct as shown in (**a**)) and miRNA mimics for 48 h, after which cell lysates were used for the luciferase assay. The data were normalized to luc activity, with the average value obtained for the p38IP 3′UTR and miR-NC (control) mimics set to 1 for (**b**). (**c**) The sequences of the predicted miR-200b-3p-binding sites on the p38IP 3′UTR in *Homo sapiens* and other species. Highly conserved nucleotides are shown in red. (**d**) Inverse correlation between p38IP mRNA and miR-200b-3p expression levels. Quantitative PCR analysis of p38IP mRNA and miR-200b-3p levels in MCF-7, U937 and 293T cells. (**e**) 293T cells were co-transfected with the wild-type or mutant p38IP 3′UTR (construct shown in (**a**)) and miR-NC (control) or miR-200b-3p mimics for 48 h. These cells were then used for luciferase reporter assays. (**f**) Western blotting analysis of p38IP protein expression in 293T cells. The cells were transfected with miR-200b-3p mimics or inhibitors for 48 h; the group with miR-NC mimics and inhibitors is indicated as the negative control. The scale bars represent the means±s.e.m. (*n*=3). **P<0.01 and ***P<0.001 compared with control groups. NS, not significant. All data are representative of at least three independent experiments with similar results.

To determine which miRNAs directly target p38IP, we conducted a preliminary luciferase reporter assay by evaluating the relative luciferase activities in 293T cells that were transfected with a reporter plasmid carrying the p38IP 3′UTR (Figure 3a) and the mimics of each of the predicted miRNAs. We found that miR-200b-3p dramatically blocked the relative activity of the reporter luciferase, thus suggesting a direct interaction with p38IP 3′UTR (Figure 3b). In addition, the binding sites of miR-200b-3p in 3′UTR of p38IP were highly conserved in *Homo sapiens* and other species (Figure 3c). Interestingly, the miR-200b-3p and p38IP mRNA expression levels were inversely correlated in different cell lines (Figure 3d), thus implying an *in vivo* relationship.

To confirm the direct interaction between miR-200b-3p and the p38IP 3′UTR, we mutated the putative interaction sites in the p38IP 3′UTR (sequences shown in Figure 3a) and found that the relative luciferase activity recovered when p38IP 3′UTR was mutated (Figure 3e). Furthermore, the level of p38IP protein expression decreased in 293T cells when they were transfected with miR-200b-3p mimics and was restored when the cells were co-transfected with miR-200b-3p inhibitors (Figure 3f). Taken together, these data indicated a direct interaction between miR-200b-3p and the p38IP 3′UTR, which allows miR-200b-3p to regulate the expression of p38IP protein.

### miR-200b-3p downregulates p38IP, promoting monocyte differentiation

**Figure 4:**
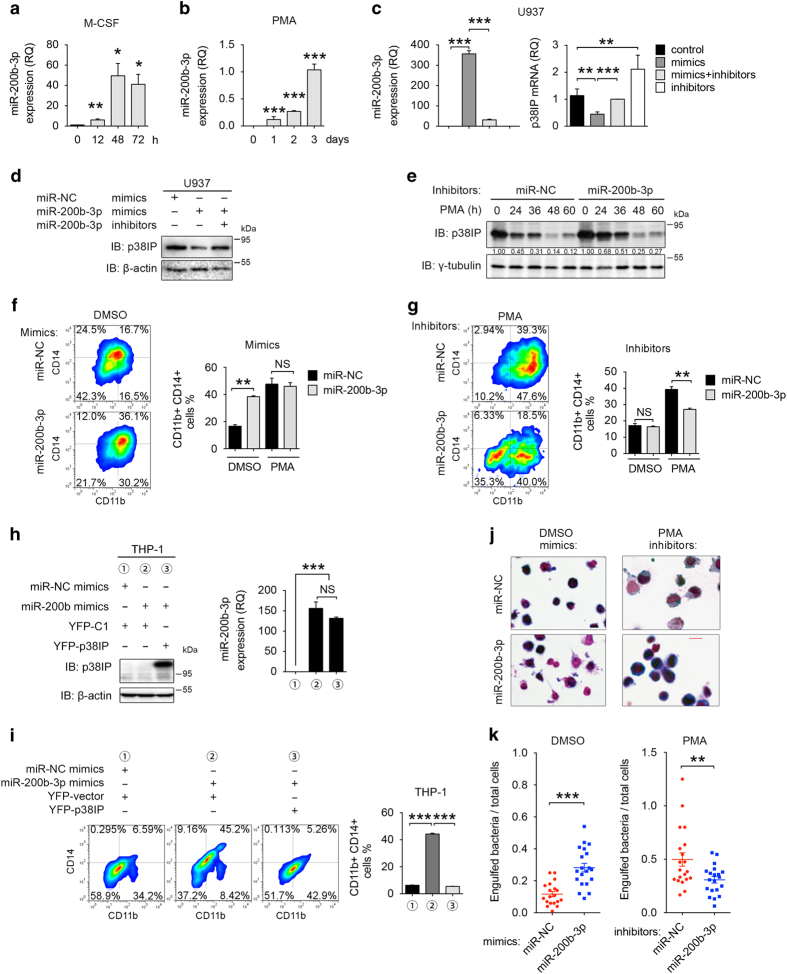
miR-200b-3p is involved in the differentiation-required p38IP downregulation. (**a**) Quantitative PCR (qPCR) analysis of miR-200b-3p levels in peripheral monocytes with macrophages colony-stimulating factor (M-CSF) stimulation for the indicated times, presented relative to U6 (short for RNU6, a commonly used normalizer for miRNA qPCR). (**b**) qPCR analysis of miR-200b-3p levels in U937 cells with phorbol myristate acetate (PMA) stimulation for the indicated times, presented relative to U6. (**c**, **d**) U937 cells were transfected with control mimics (miR-NC), miR-200b-3p mimics or inhibitors for 48 h. Then the protein and mRNA levels of p38IP were determined by qPCR (**c**) and western blotting (**d**) analysis, respectively, and miR-200b-3p expression levels were determined by qPCR analysis (**c**). (**e**) Western blotting analysis of p38IP expression in U937 cells. The cells were transfected with 200 nm miR-NC inhibitors or miR-200b-3p inhibitors and then exposed to 10 nM PMA for the indicated times. (**f**, **g**) Representative fluorescence-activated cell sorting (FACS) analysis and percent values of CD11b/CD14-positive cells are shown for the following cells and treatment conditions: U937 cells were transfected with miR-NC or miR-200b-3p mimics for 48 h (**f**), and U937 cells were transfected with miR-NC or miR-200b-3p inhibitors for 24 h and exposed to 10 nM DMSO or PMA for another 48 h (**g**). (**h**, **i**) THP-1 cells were transfected with miRNA mimics and plasmids for 24 h and exposed to 10 nM PMA for another 24 h. The expression of p38IP and miR-200b-3p was determined by western blot and qPCR, respectively (**h**). Representative FACS analysis and percent values of CD11b/CD14-positive cells are shown in (**i**). (**j**) Morphological analysis of U937 cells transfected with miRNA mimics or inhibitors. The cells were transfected for 24 h and exposed to PMA or dimethyl sulfoxide (DMSO) for another 48 h, followed by Wright–Giemsa staining. A ×630 magnification of a representative field is shown. The scale bar represents 20 μm. (**k**) Phagocytic activity of cells transfected with miRNA mimics or inhibitors. The scale bars represent the means±s.e.m. (*n*=3). *P<0.05, **P<0.01 and ***P<0.001 compared with control groups. All data are representative of at least three independent experiments with similar results. NS, not significant.

Next we examined whether miR-200b-3p is responsible for the downregulation of p38IP in the process of MO/MΦ differentiation. A significant increase in the miR-200b-3p expression levels during both M-CSF-derived human primary MO/MΦ differentiation and PMA-induced U937 differentiation was detected (Figures 4a and b) and revealed an inverse correlation between the miR-200b-3p and the p38IP protein expression levels in MO/MΦ differentiation. The expression of miR-200b-3p was also enhanced in the process of VD_3_-stimulated HL-60 cell differentiation (Supplementary Figure S3g), indicating that upregulation of miR-200b-3p is a general phenomenon in the process of differentiation. Because the miR-200 family is highly homologous in sequence and is encoded by two clusters that are located at different chromosomes (Supplementary Figures S3a and b), we examined the expression of pri-miR-200b-200a-429 and pri-miR-200c-141 clusters during M-CSF-induced monocyte differentiation and PMA-induced U937 cell differentiation. We found that the expression of pri-miR-200b-200a-429 cluster increased dramatically during differentiation; however, the expression of the pri-miR-200c-141 cluster was substantially lower (Supplementary Figures S3c and d). Consistently, the expression of mature miR-200a, miR-200b and miR-429 increased during M-CSF-treated monocyte differentiation (Supplementary Figure S3e). Further expressing miR-200b-3p mimics in U937 cells (Figure 4c) effectively reduced p38IP mRNA expression levels to 50% of normal levels (Figure 4c) and caused a downward trend in the p38IP protein levels (Figure 4d). However, expressing the miR-200a and miR-429 mimics did not decrease the expression of p38IP protein in U937 cells, although the expression of miR-429 inhibitors did indeed enhance the expression levels of p38IP protein (Supplementary Figure S3f). Interestingly, the p38IP mRNA levels in cells treated with miR-200b-3p inhibitors alone was higher than that in controls, probably owing to the inhibition of basal endogenous miR-200b-3p expression (Figure 4c). Accordingly, the p38IP protein level was increased (the 0-h point in Figure 4e). Moreover, a delay in p38IP downregulation was observed in cells that were transfected with miR-200b-3p inhibitors followed by PMA-induced differentiation (Figure 4e and Supplementary Figure S4a), suggesting that an increase in miR-200b-3p expression leads to p38IP downregulation.

Based on the above results, we next determined the role of miR-200b-3p in MO/MΦ differentiation. As expected, miR-200b-3p mimics promoted differentiation of U937 cells into macrophages (Figure 4f and Supplementary Figure S4b), whereas miR-200b-3p inhibitors suppressed U937 cells undergoing differentiation induced by PMA (Figure 4g and Supplementary Figure S4c). Similar results were obtained by using THP-1 cells (Supplementary Figure S4d). Moreover, we observed that the miR-200b-3p-promoted THP-1 differentiation was blocked by the overexpression of p38IP protein (Figures 4h and i), thus validating that the miR-200b-3p physiologically and specifically targets p38IP mRNA during MO/MΦ differentiation. In addition, U937 and THP-1 cells overexpressing miR-200b-3p mimics exhibited a more mature differentiation phenotype, whereas the cells overexpressing miR-200b-3p inhibitors maintained a typical monocyte phenotype (Figure 4j and Supplementary Figure S4e). Furthermore, after differentiation, cells transfected with miR-200b-3p mimics had greater phagocytic ability, whereas those transfected with miR-200b-3p inhibitors showed decreased phagocytic ability (Figure 4k), again verifying the critical role of miR-200b-3p/p38IP in macrophage differentiation and maturation.

### p38IP regulates G1/S phase progression

**Figure 5:**
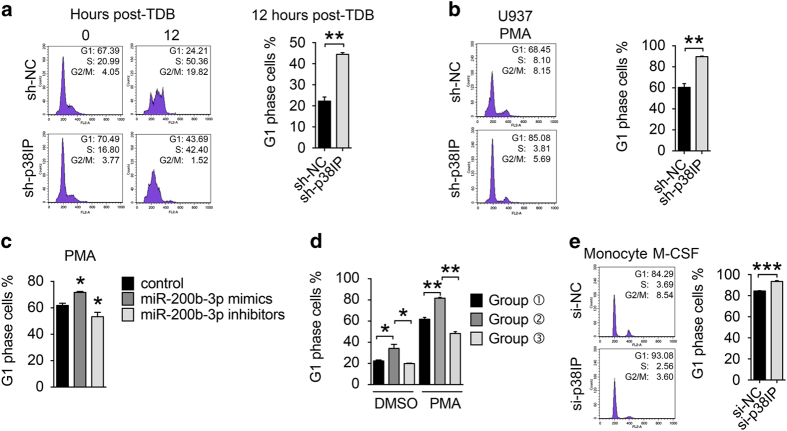
p38IP effects on cell cycle. (**a**) The cell cycle phase analysis of U937 cells transfected with sh-NC or sh-p38IP. The cells were synchronized via thymidine double block (TDB) and released for the indicated times. The percent values of cells in the G1 phase are shown at right. (**b**) The cell cycle phase analysis of sh-NC- and sh-p38IP-transfected U937 cells exposed to phorbol myristate acetate (PMA) for 48 h. The percent values of G1 phase cells are shown at right. (**c**) The percentage of G1 phase cell in U937 cells transfected with miRNA mimics or inhibitors, as shown, and exposed to PMA for 48 h. (**d**) The percentage of G1 phase cell in THP-1 cells transfected as indicated in Figure 4i and exposed to dimethyl sulfoxide (DMSO) or PMA for 24 h. (**e**) The cell cycle phase analysis of human primary monocytes. The cells were transfected with siRNAs for 48 h and then exposed to macrophages colony-stimulating factor (M-CSF) for another 3 days. The percent values of G1 phase cells are shown at right. The scale bars represent the means±s.e.m. (*n*=3). *P<0.05, **P<0.01 and ***P<0.001 compared with control groups. All data are representative of at least three independent experiments with similar results.

We next investigated the mechanism of p38IP in the regulation of differentiation. Because cell cycle exit is a necessary step in cell differentiation, we checked the effect of p38IP on cell cycle regulation during differentiation. Notably, the silencing of p38IP protein was associated with a blockade of the cell cycle in the G1 phase when U937 cells were released from double thymidine synchronization (Figure 5a). Furthermore, when U937 cells were treated with PMA to induce differentiation, a higher percentage of sh-p38IP-treated cells were blocked in G1 phase than the percentage that was blocked following sh-NC treatment (Figure 5b). In addition, it was consistently observed that the overexpression of miR-200b-3p mimics induced G1 phase arrest, whereas the overexpression of miR-200b-3p inhibitors promoted the G1–S transition (Figure 5c). Furthermore, the miR-200b-3p-promoted G1 phase arrest was rescued by the overexpression of p38IP protein especially during the PMA-induced differentiation process (Figure 5d). We also knocked down p38IP in primary human monocytes and obtained similar result (Figure 5e). These results suggest that the downregulation of p38IP by miR-200b-3p promotes MO/MΦ differentiation by halting cells in G1 phase.

### p38IP downregulation activates p38/p21 pathway

**Figure 6:**
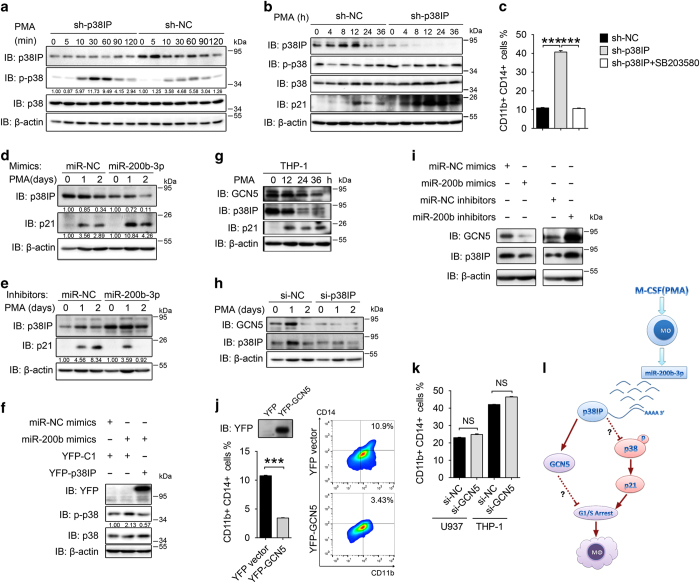
p38IP regulates cell cycle-associated proteins in a p38–p21 pathway and GCN5 cooperates with p38IP in differentiation process. (**a**) Western blotting analysis of the phosphorylation levels of p38 in U937 cells transfected with sh-NC or sh-p38IP and exposed to phorbol myristate acetate (PMA) for the indicated times. (**b**) Western blotting analysis of p-p38 and p21 in U937 cells transfected with sh-NC or sh-p38IP and exposed to PMA for the indicated times. (**c**) Fluorescence-activated cell sorting (FACS) analysis of CD11b/CD14-positive cells of sh-p38IP-transfected U937 cells treated with PMA or PMA and SB203580. (**d**) Western blotting analysis of p38IP and p21 expression in U937 cells transfected with miR-NC mimics and miR-200b-3p mimics. The cells were transfected with 200 nM mimics for 24 h and exposed to PMA for the indicated times. (**e**) Western blotting analysis of p38IP and p21 expression levels in U937 cells transfected with miR-NC or miR-200b-3p inhibitors; the cells were transfected with 200 nM inhibitors for 24 h and exposed to 10 nM PMA for the indicated times. (**f**) Western blotting analysis of p38IP expression and phosphorylation of p38 in THP-1 cells. (**g**) Western blotting analysis of GCN5 during PMA-stimulated differentiation of THP-1 cells. (**h**) Western blotting analysis of GCN5 expression in si-NC- and si-p38IP-transfected U937 cells exposed to 10 nM PMA for the indicated times; β-actin served as a loading control. (**i**) Western blotting analysis of GCN5 expression in miR-200b-3p mimic- or inhibitor-transfected U937 cells; β-actin served as a loading control. (**j**) GCN5 was overexpressed in THP-1 cells for 48 h, and the cells were exposed to PMA to induce differentiation. The overexpression of GCN5 was verified by western blotting, and CD11b/CD14-positive cells were detected by flow cytometry. Representative FACS analysis and percent values of CD11b/CD14-positive cells are shown. (**k**) The percent values of CD11b/CD14-positive cells in U937 and THP-1 cells after knockdown of GCN5 for 48 h followed by exposing to 10 nM PMA for another 48 h. All data are representative of at least three independent experiments with similar results. The scale bars represent the means±s.e.m. (*n*=3). NS, not significant. ***P<0.001 compared with control groups. Green fluorescent protein antibody was used for detection of yellow fluorescent protein (YFP)-fused proteins, because it is also reactive against YFP. (**l**) The model for the function of p38IP in the regulation of monocyte/macrophage (MO/MΦ) differentiation. IB, immunoblotting.

Cyclin-dependent kinase inhibitors, particularly p21, are well-known cell differentiation inducers that promote cell cycle exit in G1 phase [28], and the upregulation of p21 promotes MO/MΦ differentiation [29, 30]. Furthermore, p38IP interacts with p38, and p38 is involved in the G1/S checkpoint by regulating p21 [31–33]. Therefore, we analyzed p38 activation and p21 expression in p38IP-regulated MO/MΦ differentiation. We found that upon short-term PMA stimulation of U937 cells, the phosphorylation of p38 was enhanced when p38IP was knocked down (Figure 6a). Next we determined the phosphorylation levels of p38 and the accumulation of p21 in U937 cells by long-term PMA stimulation and found that during MO/MΦ differentiation, the phosphorylation of p38 was stronger and steadier when p38IP was knocked down (Figure 6b). Further, the accumulation of p21 protein was accelerated (Figure 6b). To elucidate whether p38IP regulates MO/MΦ differentiation by controlling the p38 activity, we blocked p38 activity by using its specific inhibitor SB203580 and found that knockdown of p38IP-induced MO/MΦ differentiation was significantly decreased (Figure 6c). The above results suggested that downregulation of p38IP could promote the activation of p38 and, in turn, the accumulation of p21, ultimately leading to G1/S arrest and MO/MΦ differentiation.

Similarly, during MO/MΦ differentiation, p21 protein accumulated more intensely when p38IP expression was downregulated by miR-200b-3p mimics (Figure 6d); conversely, the accumulation of p21 protein was much weaker and unsteady when p38IP expression was stabilized by miR-200b-3p inhibitors (Figure 6e). Furthermore, overexpression of miR-200b-3p enhanced p38 phosphorylation, which could be restored by p38IP overexpression (Figure 6f). Taken together, these results suggest that miR-200b-3p promotes MO/MΦ differentiation by specifically targeting p38IP.

### GCN5 is involved in p38IP-regulated MO/MΦ differentiation

As a subunit of the GCN5/SAGA acetyltransferase complex, p38IP stabilizes the GCN5 protein [8]. In addition, GCN5 has been reported to regulate G1/S progression [34, 35] and macrophage differentiation [36]. Thus we analyzed whether GCN5 was involved in p38IP downregulation-induced macrophage differentiation. We observed a decrease in GCN5 expression in addition to p38IP downregulation and p21 accumulation during the differentiation (Figure 6g). Furthermore, the knockdown of p38IP by si-p38IP accelerated the decrease of GCN5 protein levels in PMA-induced differentiation (Figure 6h), which is in line with our report that p38IP stabilizes GCN5 protein [8]. In addition, the miR-200b-3p mimic-induced downregulation of p38IP decreased GCN5 protein levels, whereas the expression of miR-200b-3p inhibitor-induced increase in p38IP protein levels resulted in a stabilization of the GCN5 protein levels (Figure 6i). Suspecting that GCN5 downregulation is required for the p38IP/p38/p21 axis-controlled MO/MΦ differentiation, we ectopically expressed GCN5 in THP-1 cells and found that it blocked PMA-induced differentiation similarly to p38IP (Figure 6j). Surprisingly, the knockdown of GCN5 by siRNAs did not accelerate MO/MΦ differentiation (Figure 6k). Taken together, these results suggest that GCN5 downregulation is essential but not sufficient for MO/MΦ differentiation and indicate that p38IP regulates MO/MΦ differentiation by integrating both GCN5 downregulation and p38/p21 signaling.

Finally, to probe whether our findings could reflect *in vivo* MO/MΦ differentiation, we analyzed data from the ONCOMINE database. We found that the expression of p38IP increased up to 2.5-fold in leukemia samples (7347 samples from 81 leukemia data sets) compared with normal samples (data not shown), thus further implying the importance of p38IP in hematopoiesis, particularly in MO/MΦ differentiation.

## Discussion

Here we identified a novel miR-200b-3p/p38IP regulatory pair in MO/MΦ differentiation. We demonstrated that upon M-CSF or PMA stimulation, p38IP in monocyte was downregulated by the upregulated miR-200b-3p, which promoted p38 activation and p21 accumulation and, in turn, led to G1/S arrest and MO/MΦ differentiation. The p38IP downregulation-induced decline of GCN5 was also involved in the differentiation. Our findings not only uncovered a novel miRNA/target pair and signaling axis for MO/MΦ differentiation but also revealed an unknown function of p38IP (Figure 6l).

MO/MΦ differentiation is a complex process involving the participation of various factors. miR-424 and miR-155 have been widely demonstrated to be involved in the regulation of MO/MΦ differentiation [37], and they were predicted to target p38IP using bioinformatics tools. However, our data showed that miR-424 or miR-155 had no effect on p38IP, thus providing further insight into the specificity of miRNA regulation and the existence of multiple regulatory mechanisms in the process of differentiation. Our discovery of the essential role of the miR-200b-3p/p38IP pair in monocyte differentiation further expands the function of the miR-200 family beyond tumor-suppressive signatures [38] and apoptosis [39–41]. In response to different stimuli, macrophages may polarize to M1 (classically activated macrophages) and M2 (alternatively activated macrophages), which have distinct and somewhat opposite effector functions [42]. Whether the miR-200b-3p/p38IP pair has a role in macrophage polarization represents an intriguing future aim.

The involvement of p38IP or miR-200b-3p in EMT has been reported. During the gastrulation stage of mouse embryo development, p38IP is critically required for the downregulation of E-cadherin to promote EMT [3]. In addition, miR-200 family members have been demonstrated to function as negative regulators in EMT [43–47]. The opposing functions of p38IP and miR-200b-3p in the regulation of EMT and our demonstration of the interaction between p38IP and miR-200b-3p in MO/MΦ differentiation suggest that these two molecules might also function as a miRNA/target pair in EMT progress. Moreover, EMT has recently been reported to be related to a stem cell-like phenotype [48, 49], and the miR-200 family was described to decrease stem-like properties and promote the transition from stem-like to non-stem phenotypes [50, 51]. p38IP was also predicted to be a novel transcription factor in human CD34 antigen-positive hematopoietic stem cells [1]. Therefore, miR-200b-3p/p38IP pair may also have an important role in the regulation of hematopoietic stem cell properties and other stages of hematopoiesis, in addition to MO/MΦ differentiation.

p38 is also known to be involved in cell differentiation for certain cell types [52], for example, erythroid differentiation [53–56], osteoblast differentiation [57], myogenic differentiation [58] and monocyte differentiation [59, 60]. Our finding that p38 activation is required for miR-200b-3p/p38IP-mediated monocyte differentiation supplies new evidence for its importance in differentiation. The miR-200b-3p/p38IP pair might be involved in the activation of p38 during the differentiation of other cell types. Of note, our study demonstrated that the downregulation of p38IP activates p38, whereas in the study of EMT, p38IP was found to bind directly to p38 and activated p38 *in vivo* during mice gastrulation [3]. The opposing effects of p38IP on the activation of p38 could result from different signaling contexts, thus reflecting the flexibility of p38IP regulation.

In this study, we have shown that p38IP controls MO/MΦ differentiation through regulation of GCN5 acetyltransferase protein expression levels. Epigenetic modification involving histone acetylation has important role in controlling myeloid cell differentiation [61]. For example, transcription of the histone deacetylase 1 gene was repressed during myeloid differentiation [62], and changes in H3K27 acetylation closely correlate with the MO/MΦ differentiation process and thus function as a MO/MΦ differentiation marker [63]. As a histone acetyltransferase, GCN5 can acetylate histones H3 and H4 at specific lysines and preferentially modifies histone H3 on lys9 and lys14 [64]. Our study showed that the decline of GCN5 is required for miR-200b-3p/p38IP-regulated MO/MΦ differentiation, which implies that the declines of GCN5-mediated histones acetylations might be involved in MO/MΦ differentiation. In addition, unlike its negative role in MO/MΦ differentiation, GCN5 generally has positive regulatory roles in muscle cell, preadipocyte and cardiomyocyte differentiation [65–67]. These differing roles indicate that differentiation modules are cell specific.

In summary, we have identified a novel pathway in which the downregulation of p38IP by miR-200b-3p promotes MO/MΦ differentiation by activating p38 and causing an accumulation of p21. Our discovery not only improves our understanding of monocyte/macrophage differentiation but also provides a potential strategy for leukemia treatment.

## Materials and Methods

### Antibodies and reagents

Thymidine, PMA and propidium iodide were purchased from Sigma-Aldrich (St Louis, MO, USA); FAM48A (p38IP), p38, p21, GCN5, green fluorescent protein (GFP), γ-Tubulin, β-actin and horseradish peroxidase-conjugated secondary antibodies were purchased from Santa Cruz Biotechnology (Santa Cruz, CA, USA); anti-Phospho-p38 mitogen-activated protein kinase (Thr180/Tyr182) was purchased from Cell Signaling Technology (Danvers, MA, USA); PE Mouse anti-human CD11b/Mac-1 and APC Mouse anti-human CD14 were purchased from BD Pharmingen (San Diego, CA, USA); CD14+ microbeads (human) was purchased from Miltenyi Biotec, Bergisch Gladbach, Germany; Recombinant Human M-CSF (cyt-308) and murine M-CSF was purchased from Prospec protein specialists (East Brunswick, NJ, USA). As GFP antibody is also reactive against yellow fluorescent protein, it was used for detection of yellow fluorescent protein.

### Cell culture, transfections and differentiation

U397, THP-1 and HL-60 cells were cultured in RPMI-1640 medium, and HEK-293T cells were cultured in Dulbecco's modified Eagle's medium (Invitrogen, Carlsbad, CA, USA). Both cultures were supplemented with 10% (v/v) fetal bovine serum (Hyclone, Logan, UT, USA). Peripheral blood mononuclear cells obtained from healthy donors were isolated by centrifugation over Ficoll-Hypaque (Hao Yang Co., Guangzhou, China) according to the manufacturer's instructions. Human peripheral blood monocytes were purified using CD14+ microbeads according to the manufacturer’s instructions (Miltenyi Biotec). For differentiation, U937, THP-1 and HL-60 cells (3×10^5^ cells ml^−1^) were cultured in the presence of PMA (10 nm), VD_3_ (50 nM) or ATRA (1 μM), and monocytes (5×10^5^ cells ml^−1^) were cultured in the presence of recombinant M-CSF (50 ng ml^−1^) for the indicated times. U937 and THP-1 cells were transfected with shRNAs or siRNAs using Lipofectamine LTX reagent (Invitrogen). For miRNA mimic or inhibitor transfection, U937 and THP-1 cells were nucleoporated using the Amaxa Nucleofector Kit following the manufacturer's instructions (Lonza, Basel, Switzerland); 293T cells were transfected using Lipofectamine 2000 reagent (Invitrogen).

### Plasmids, shRNAs, siRNAs, miRNA mimics and inhibitors

The 3′UTR of p38IP (approximately 400 bp, Accession NM_017569.3) was amplified from U937 cell cDNA and inserted into the psiCHECK2 vector (Promega, Madison, WI, USA) for the luciferase assay; the p38IP 3′UTR mutation was mutated using pfuUltrlII DNA polymerase according to the manufacturer's instructions (Stratagene, San Diego, CA, USA). The p38IP shRNA sequence was designed according to the Invitrogen Block-iT RNAi Designer and synthesized by BioSune Biotechnology Co. Ltd (Guangzhou, China). Scrambled control nucleotides (5′-CGCTAATTCGACTCGGATA-3′) and sh-p38IP sequence (5′-ACACAAGAGCACTGAATCA-3′) were constructed into RNA interference expression vector pSUPER.Retro.Neo-GFP (OligoEngine, Seattle, WA, USA), respectively. The scrambled control siRNA, including si-p38IP-1 (5′-GCTTGTTATGCAAGAGACT-3′), si-p38IP-2 (5′-GCAACAAGCTTTAGAACTA-3′) and si-GCN5 (5′-GGAAAUGCAUCCUGCAGAUdtdT-3′), were synthesized by RiboBio Co. Ltd (Guangzhou, China). miRNA mimics and inhibitors were purchased from RiboBio Co. Ltd.

### Western blotting analysis

Western blotting analysis was performed as previously described [68]. Briefly, Cells were lysed in buffer (20 mM Tris-HCl, pH 7.5, 150 mM NaCl, 5 mM EDTA, 1% Nonidet P40 or 1% digitonin (D141; Sigma, St Louis, MO, USA)) supplemented with protease inhibitors (10 μg ml^−1^ aprotinin, 10 μg ml^−1^ leupeptin and 1 mm phenylmethanesulfonylfluoride) and phosphatase inhibitors (5 mM sodium pyrophosphate and 1 mm Na_3_VO_4_). Whole-cell lysates were resolved by sodium dodecyl sulfate-polyacrylamide gel electrophoresis, transferred onto a polyvinylidene difluoride membrane and probed overnight at 4 °C with primary antibodies, followed by incubation for 1 h at room temperature with horseradish peroxidase-conjugated secondary antibodies. Signals were visualized by enhanced chemiluminescence (GE Healthcare, Little Chalfont, UK) and exposed to X-ray film or on the ChemiDoc XRS+ system (Bio-Rad, Hercules, CA, USA). Densitometry analysis was performed with the ImageJ software (Bethesda, MD, USA).

### RNA isolation and real-time PCR

Total RNAs were extracted with Trizol (Invitrogen) and reverse-transcribed into cDNAs followed by real-time PCR using SYBR Green (Roche, Indianapolis, IN, USA). Specifically, miRNAs were precipitated in isopropanol and placed in −80 °C freezer overnight and reverse transcribed with specific Bulge-Loop RT primers (RiboBio Co. Ltd). Relative quantitative RNA was normalized with GAPDH (glyceraldehyde 3-phosphate dehydrogenase; for p38IP) or U6 small nuclear RNA (for miR-200b-3p). The following oligonucleotides were used for quantitative PCR: p38IP (sense, 5′-TGGCAAACTCTGCTGGACTT-3′, antisense, 5′-TTGAACCTTGCTCAGAACCCT-3′) and GAPDH (sense, 5′-ACGGATTTGGTCGTATTGGG-3′, antisense, 5′-TGATTTTGGAGGGATCTCGC-3′); miRNAs and U6 small nuclear RNA primers were synthesized by RiboBio Co. Ltd. All of these reactions were performed in triplicate.

### Luciferase assay

Transient transfections were performed in HEK-293T cells in 96-well plates with 100 nM miR-200b-3p or miR-NC mimic and 10 ng of psiCHECK2-p38IP 3′UTR wild type or mutation. After 48 h, cells were collected in 50 μl reporter lysis buffer, and 5 μl of lysate were assayed for luciferase activity by Bioluminometer (Berthold, Bad Wildbad, Germany) according to the manufacturer's protocols. Each transfection was carried out in triplicate.

### Flow cytometry

At the indicated times, cells were processed for double staining (30 min at 4 °C) using PE-CD11b and APC-CD14. For cell cycle analysis, cells were fixed with 70% ethanol overnight and stained with propidium iodide for 30 min at room temperature. Fluorescence acquisition was carried out on fluorescence-activated cell sorter calibur (BD, Franklin Lakes, NJ, USA), and data analysis was carried out using the Cellquest (Franklin Lakes, NJ, USA), Flowjo (Ashland, OR, USA), ModFit LT (Topsham, ME, USA) and GraphPad prism software.

### Phagocytic assay

To evaluate the phagocytic activity, U937 cells were cultured in a 24-well plate with glasses and exposed to 10 nM PMA for 48 h and then incubated with mcherry-conjugated *E. coli* in fresh medium at 37 °C for 1 h. A negative control was performed by incubating cells with *E. coli* on ice. After incubation, cells were immediately put on ice to stop phagocytosis and washed by cold phosphate-buffered saline for three times. The phagocytosis glasses were examined by microscopy (magnification at ×630).

### Statistical analysis

All analyses were performed using GraphPad Prism version 5.0 (GraphPad Software, La Jolla, CA, USA). The data are presented as the means±s.e.m., unless otherwise stated. The statistical significance of differences between two groups was assessed by unpaired Student's *t*-tests, and *P*-value is shown in the figures.
