# Supplementary material for: A novel miR-200b-3p/p38IP pair regulates monocyte/macrophage differentiation
Source: Cell Discov. 2016 Jan 26;2:15043–. doi: 10.1038/celldisc.2015.43 (PMC4860955; doi:10.1038/celldisc.2015.43)
Supplement: Supplementary Figure S1 [file celldisc201543-s1.pdf]

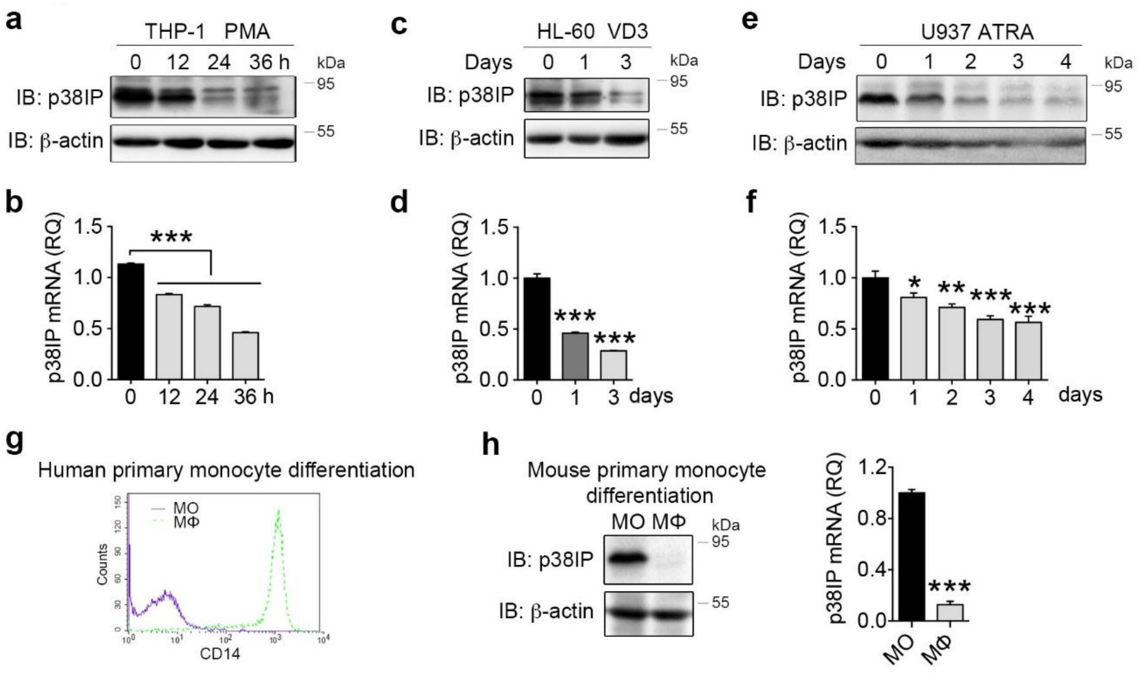


**Supplementary Figure S1** Downregulation of p38IP during differentiation. (**a**-**b**) Time-dependent downregulation of p38IP in THP-1 cells with PMA stimulation. THP-1 cells were treated with PMA for the indicated times to induce differentiation. The cells were collected for western blot analysis of p38IP protein expression (**a**) or qPCR analysis of p38IP mRNA expression (**b**). (**c**-**d**) Time-dependent downregulation of p38IP in HL-60 cells with $VD_3$ stimulation. HL-60 cells were treated with $VD_3$ for the indicated times to induce differentiation. The cells were collected for western blot analysis of p38IP protein expression (**c**) or qPCR analysis of p38IP mRNA expression (**d**). (**e**-**f**) Time-dependent downregulation of p38IP in U937 cells with ATRA stimulation. U937 cells were treated with ATRA for the indicated times to induce differentiation. The cells were collected for western blot analysis of p38IP protein expression (**e**) or qPCR analysis of p38IP mRNA expression (**f**). (**g**) Human primary monocytes were differentiated into macrophages by culturing with recombinant

human M-CSF for 7 days. Cells were harvest and subjected to FACS analysis of CD14 expression. (**h**) Mouse primary monocytes were differentiated into macrophages by culturing with recombinant murine M-CSF for 7 days, the cell lysates were used for western blot analysis of p38IP protein expression and qPCR analysis of p38IP mRNA expression. The scale bars represent the means ± standard error of mean (SEM) (n=3). * $P<0.05$, ** $P<0.01$, *** $P<0.001$, versus controls. The data are representative of at least three independent experiments with similar results.
