# Supplementary material for: A novel miR-200b-3p/p38IP pair regulates monocyte/macrophage differentiation
Source: Cell Discov. 2016 Jan 26;2:15043–. doi: 10.1038/celldisc.2015.43 (PMC4860955; doi:10.1038/celldisc.2015.43)
Supplement: Supplementary Figure S2 [file celldisc201543-s2.pdf]

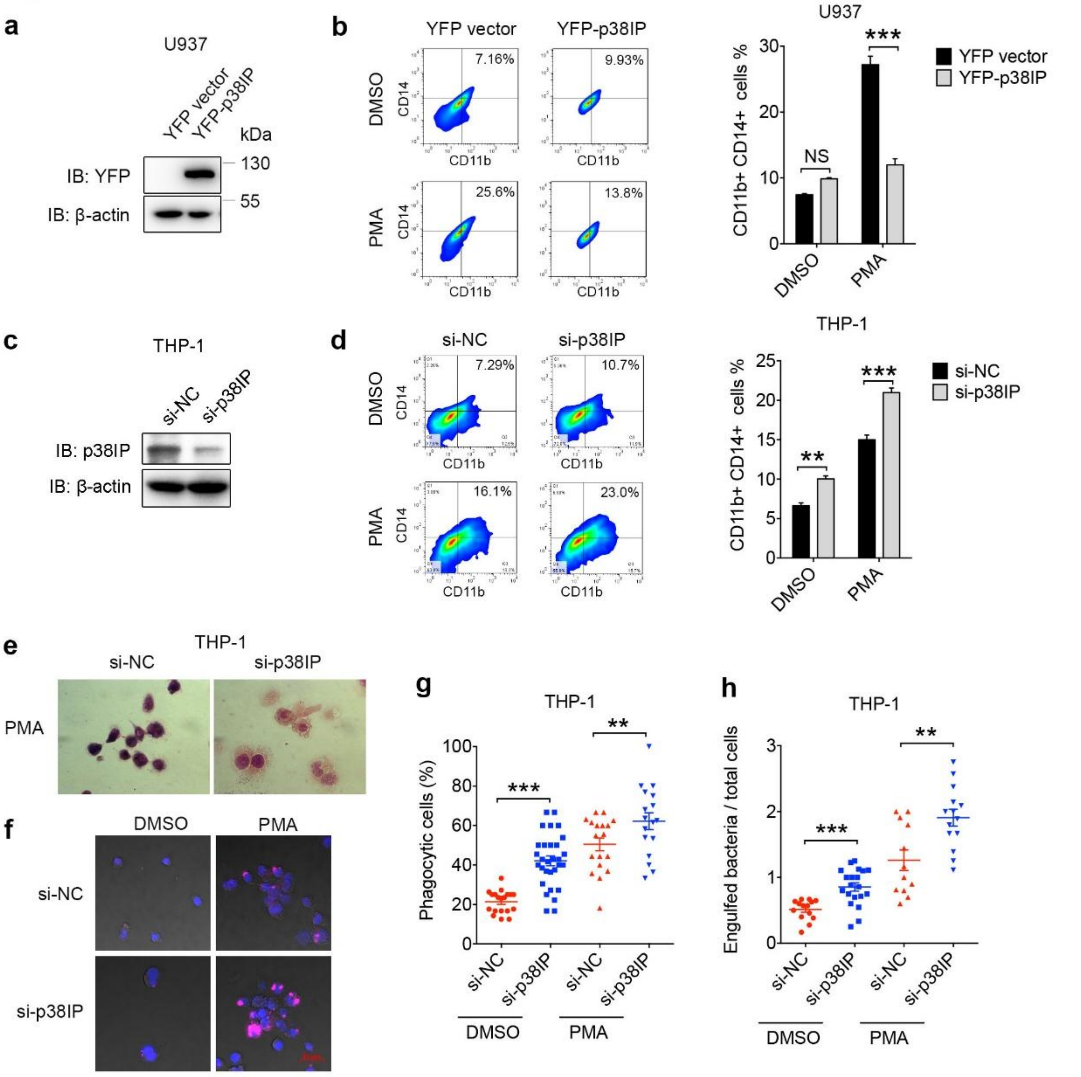

Supplementary Figure S2 p38IP negatively regulates U937 and THP-1 cells differentiation and function. (**a**-**b**) U937 cells were transfected YFP-tagged p38IP or empty vector, followed by PMA stimulation. The overexpression of p38IP was verified by western blot (**a**), and CD11b/CD14-positive cells were detected by flow cytometry. Representative FACS analysis and percent values of CD11b/CD14-positive cells are shown in (**b**). (**c**-**d**) p38IP was knocked down by siRNAs in THP-1 cells for 48 h, and the cells were then stimulated with PMA (DMSO serves as control) for 48 h. The knockdown efficiency was determined by western blot (**c**), and CD11b/CD14-positive

cells were detected by flow cytometry. Representative FACS analysis and percent values of CD11b/CD14-positive cells are shown (**d**). (**e**) Morphological analysis of THP-1 sI-NC and sI-p38IP cells. The cells were exposed to PMA for 48 h and then stained by Wright-Giemsa staining. A×630 magnification of a representative field is shown. The scale bar represents 20 μm. (**f**-**h**) THP-1 cells (si-NC, si-p38IP) were exposed to 10 nM PMA for 48 h (DMSO treatment serves as a negative control) and incubated with labeled *E.coli* bacteria for 1 h. A representative field of phagocytic activity is shown in (**f**), The scale bar represents 20 μm. Twenty view fields of view were selected randomly, and both phagocytic and total cells were counted. The ratio was measured and is shown in (**g**). Engulfed bacteria and total cells were counted, and the ratio is shown in (**h**). The scale bars represent the means ± standard error of mean (SEM) (n=3). ** $P<0.01$, *** $P<0.001$ compared with control groups. All data are representative of at least three independent experiments with similar results.
