# Supplementary material for: A novel miR-200b-3p/p38IP pair regulates monocyte/macrophage differentiation
Source: Cell Discov. 2016 Jan 26;2:15043–. doi: 10.1038/celldisc.2015.43 (PMC4860955; doi:10.1038/celldisc.2015.43)
Supplement: Supplementary Figure S3 [file celldisc201543-s3.pdf]

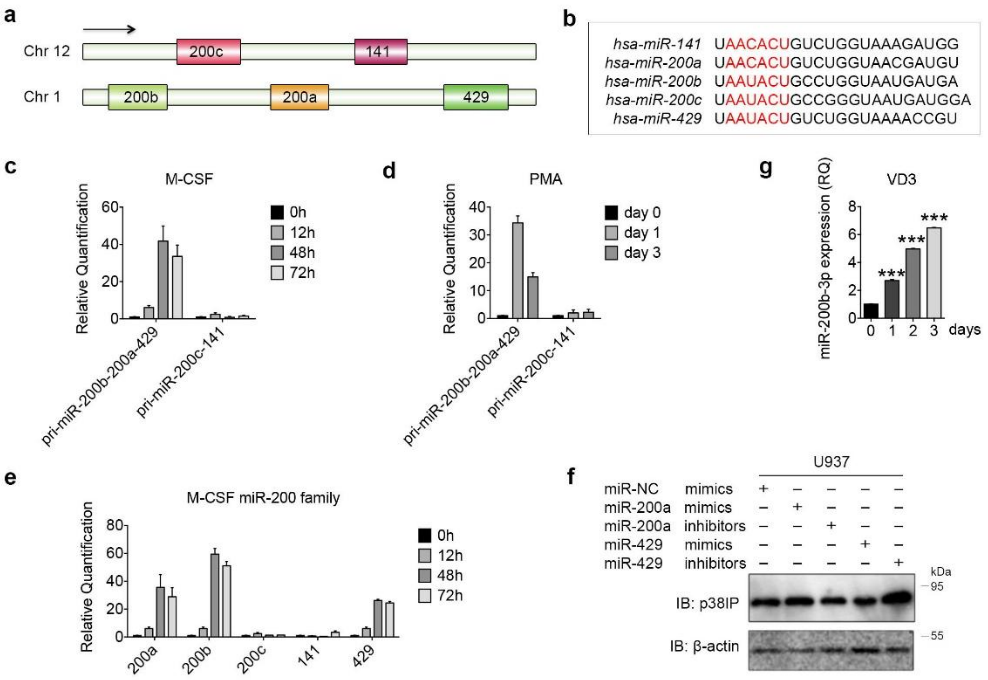

Supplementary Figure S3 Expression of miR-200 family during monocyte/macrophage differentiation. (**a**) Schematic representation of the genomic localization of the two clusters containing the miR-200 family members. (**b**) Sequence of the five members of miR-200 family. The seed sequence is highlighted in red. (**c**) qPCR analysis of pri-miR-200b-200a-429 and pri-miR-200c-141 levels in peripheral monocytes with M-CSF stimulation for the indicated times. (**d**) qPCR analysis of pri-miR-200b-200a-429 and pri-miR-200c-141 levels in U937 cells with PMA stimulation for the indicated times. (**e**) qPCR analysis of miR-200a, miR-200b, miR-200c, miR-141 and miR-429 levels in peripheral monocytes with M-CSF stimulation for the indicated times. (**f**) U937 cells were transfected with control mimics (miR-NC), mimics or inhibitors of miR-200a or miR-429 for 48 h. Then, the protein levels of p38IP were determined by western blot analysis. (**g**) qPCR analysis of miR-200b-3p levels in HL-60 cells with VD_3 stimulation for the indicated times,

presented relative to U6. All data are representative of at least three independent experiments with similar results.
