# Supplementary material for: A novel miR-200b-3p/p38IP pair regulates monocyte/macrophage differentiation
Source: Cell Discov. 2016 Jan 26;2:15043–. doi: 10.1038/celldisc.2015.43 (PMC4860955; doi:10.1038/celldisc.2015.43)
Supplement: Supplementary Figure S4 [file celldisc201543-s4.pdf]

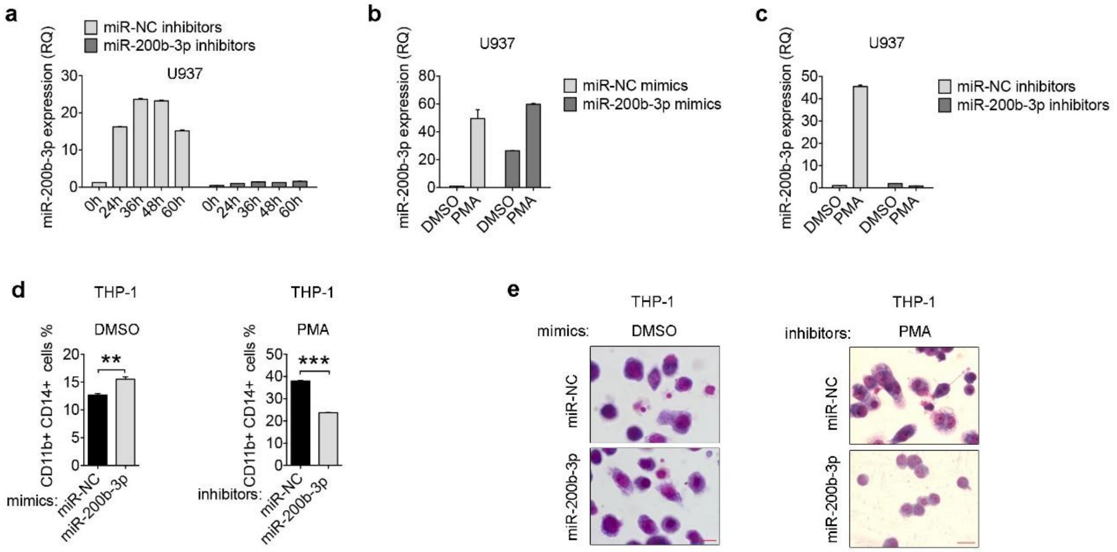

Supplementary Figure S4 miR-200b-3p is involved in the differentiation-required p38IP downregulation.

(**a**) U937 cells were transfected with miR-NC inhibitors or miR-200b-3p inhibitors, then exposed to PMA for the indicated times, and miR-200b-3p expression level was determined by qPCR analysis. (**b**-**c**) U937 cells were transfected with miR-NC or miR-200b-3p mimics (**b**) or inhibitors (**c**), then exposed to PMA (DMSO serves as control) for 2 days, and miR-200b-3p expression level was determined by qPCR analysis. (**d**) Percent values of CD11b/CD14-positive cells are shown of THP-1 cells with the following treatment: cells were transfected with miRNA mimics or inhibitors for 24 h, then exposed to DMSO or PMA as indicated for another 48 h. (**e**) Morphological analysis of THP-1 cells transfected with miRNA mimics or inhibitors. The cells were transfected for 24 h and exposed to DMSO or PMA as indicated for another 48 h, followed by Wright-Giemsa staining. A×630 magnification of a representative field is shown. The scale bar represents 20 μm. The scale bars represent the means ± standard error of mean (SEM) (n=3). ** $P<0.01$ and *** $P<0.001$ compared with control groups. All data are representative of at least three independent experiments with similar results.
